# Spatio-temporal trends in mortality due to Chagas disease in the State of Bahia, Brazil, from 2008 to 2018

**DOI:** 10.1590/0037-8682-0058-2024

**Published:** 2024-10-28

**Authors:** Cristiane Medeiros Moraes de Carvalho, Gilmar Ribeiro-Jr, Rodrigo Gurgel-Gonçalves, Liane Santiago Andrade, Cicílio Alves Moraes, Maria Aparecida Araújo Figueiredo

**Affiliations:** 1 Universidade do Estado da Bahia, Departamento de Ciências da Vida, Programa de Pós-Graduação em Saúde Coletiva, Salvador, BA, Brasil.; 2 Diretoria de Vigilância Epidemiológica, Secretaria da Saúde do Estado da Bahia, Salvador, BA, Brasil.; 3 Instituto Gonçalo Moniz - FIOCRUZ-BA, Laboratório de Patologia e Biologia Molecular, Salvador, BA, Brasil.; 4 Fundação Oswaldo Cruz - FIOCRUZ-RJ, Programa de Pesquisa Translacional em Doença de Chagas (Fio-Chagas), Rio de Janeiro, RJ, Brasil.; 5 Universidade de Brasília, Faculdade de Medicina, Laboratório de Parasitologia Médica e Biologia Vetores, Brasília, DF, Brasil.; 6 Universidade Federal de Goiás, Centro de Estudos da Doença de Chagas, Hospital das Clínicas, Goiânia, GO, Brasil.

**Keywords:** Chagas Disease, Neglected Tropical Diseases, Spatio-Temporal Analysis, Mortality, Health Regionalization

## Abstract

**Background:**

Chagas disease is a silent illness with high mortality burden in many Latin American countries, such as Brazil. Bahia has the fourth highest mortality rate in Brazil. This study analyzed the temporal trends and regional differences in the mortality rate of Chagas disease in Bahia State from 2008 to 2018.

**Methods:**

A time-series analysis of Chagas disease-related deaths was conducted using data from the Mortality Information System of Brazil. We compared the mortality rate due to Chagas disease as the primary cause and mention of the disease in the death certificate, standardized by age and health macroregion/residence municipality, and mapped hot and coldspots.

**Results:**

The Chagas Disease Mortality Rate in Bahia during the study period revealed a stationary trend, ranging from 5.34 (2008) to 5.33 (2018) deaths per 100,000 inhabitants. However, the four health macroregions showed an upward trend in mortality rates. The mortality rate (age-adjusted) ranged from 4.3 to 5.1 deaths per 100,000 inhabitants between 2008 and 2018. We observed a upward trend in the mortality rate among individuals aged ≥70 years and a higher incidence of death among men than among women. Of the total number of deaths (8,834), 79.3% had Chagas disease as the primary cause and the death certificates of 20.7% mentioned the disease. Cardiac complications were reported in 85.1% of the deaths due to Chagas disease.

**Conclusions:**

The regional and individual differences in the mortality rate of Chagas disease highlighted in this study may support health planning that considers the peculiarities of the territory.

## INTRODUCTION

Chagas disease (CD) is an infectious disease caused by the protozoan *Trypanosoma cruzi*. It is transmitted naturally by blood-sucking triatomine bugs (Hemiptera: Reduviidae)^1^, via food or beverages contaminated with the parasite, vertical transmission, and blood. This condition primarily affects socially vulnerable populations and can lead to severe cases of the disease and death, impacting the health care and social security systems of a country^2^. Due to the limited resources available for research, management, diagnosis, and treatment, CD is classified as a neglected tropical disease (NTD)^3^.

Approximately 6-8 million individuals are predicted to be infected with *T. cruzi* in the Americas, and the rate of congenital transmission of *T. cruzi* infection is 2-8%, resulting in approximately 8,000 infected newborns each year^4^. At least 5 million individuals in Latin America carry *T. cruzi,* and approximately 30-40% of them have developed or will develop cardiomyopathy, digestive disorders, or other clinical complications^5^. Owing to the absence of symptoms, only seven of every 10 infected individuals are diagnosed and only 1% receive treatment, with an expected number of deaths exceeding 10,000 annually^4^.

The mortality rate due to Chagas disease (MRCD) is high in Brazil, accounting for 74.9% of deaths attributed to NTD between 2000 and 2019^6^. Although a downward trend in MRCD was observed in Brazil, some states, such as Goiás, Minas Gerais, Bahia, and Tocantins, exhibited different patterns, with the highest MRCD occurring in some periods^7^.

From an entomological perspective, recent research has shown the widespread distribution of triatomines in Bahia, with *T. cruzi* infection rates of approximately 10%. Blood meal analysis revealed human and domestic animal DNA within the guts of triatomines, indicating the potential risk of vector transmission^8^. Ribeiro-Jr et al.^9^ suggested that native triatomines in Bahia maintain the risk of *T. cruzi* transmission in approximately 76% of the municipalities. Therefore, we believe that the CD mortality trends in Bahia are heterogeneous and that a higher MRCD is observed in areas at risk of *T. cruzi* vector transmission. From the perspective of health surveillance, despite the existence of well-established hemovigilance in the country^10^, other factors, such as low suspicion and lack of knowledge among health professionals and the population about the disease, may contribute to the increase in MRCD, which increases the difficulty of identifying the most vulnerable regions for CD in the country^11^. 

This study aimed to analyze the temporal trends and regional differences in MRCD in Bahia State from 2008 to 2018.

## METHODS

We conducted a time-series study of MRCD in Bahia State, Northeast Brazil, between 2008 and 2018. Bahia covers an area of 564,760.427 square kilometers, with an estimated population of 14,930,634 in 2020^12^. The state has 417 municipalities, 28 health regions, and nine health macroregions^13^ (Supplementary Figure 1).

The study population consisted of individuals who died from CD (primary cause/mention of the disease) between 2008 and 2018 and were registered in the Mortality Information System (SIM) of Brazil¹⁴. We used the following codes from the International Classification of Diseases, Tenth Revision (ICD-10): B57.0, acute form of CD with cardiac involvement; B57.1, acute form of CD without cardiac involvement; B57.2, chronic CD with cardiac involvement; B57.3, chronic CD with digestive system involvement; B57.4, chronic CD with nervous system involvement; B57.5, chronic CD with involvement of other organs; K23.1, megaesophagus in CD; K93.1, megacolon in CD (17); O00-O99, maternal deaths and B94.8, sequelae from infectious diseases. Additionally, we included reports mentioning any of the above codes.

According to the World Health Organization, we considered the primary cause “a disease or injury that initiates the chain of pathological events leading directly to death”¹⁵. Death due to CD was identified when another disease was selected as the primary cause, which allowed us to identify a larger number of *T. cruzi-*infected individuals who died. These data were individually reviewed to correct errors that could have led to bias in the analysis. We considered the following independent variables in the analysis: years (2008-2018), age group (≤29; 30-39; 40-49; 50-59; 60-69; 70-79; ≥80 years), sex (men/women), health macroregions/health regions and municipality of residence.

The database of CD-related deaths in Bahia originally contained 8,834 records based on the primary cause or mention of CD between 2008 and 2018. However, 28 of these records did not specify the municipality of residence, and 25 had an invalid code (B57) as the primary cause or mention of CD. Deaths for which residential information was lacking were excluded from the analysis. 

For the annual trend analysis of MRCD, we constructed line graphs displaying the raw coefficient (Brazil) and age-standardized rate (Bahia) data organized by the year of occurrence. Rates were standardized according to Brazilian population data segmented by age group and year^12^ ^;^ ^14^ ^;^ ^16^ ^-^ ^17^. 

The mortality rate was the number of deaths recorded in the SIM by year in individuals residing in cities located in Bahia State divided by the population at risk in the same year and place per 100,000 inhabitants. The mortality rate by cause was death due to CD (underlying cause or mention of CD), recorded in SIM, by place of residence (macroregion or municipality), according to the year of occurrence of death, divided by the population of the same place and period, and multiplied by 100,000.

We examined the distribution of MRCD, categorized by primary cause or mention of CD, based on the health macroregion of residence using boxplots arranged in descending order from the median^18^. To calculate medians and construct boxplots, we assumed that municipalities with an MRCD value of “zero” did not report CD-related deaths, as the absence of CD-related deaths cannot be guaranteed in these areas. 

Additionally, we calculated the MRCDs and mapped them according to municipalities and health regions. We used ArcMap® 10.5 (Redlands, California) and QGIS 3.34 Prizren software for visualization, analysis, and determination of geospatial clustering patterns in the data. In the maps, the municipal MRDC values were categorized into 10 classes according to the quantiles of data distribution. To identify hotspots, we utilized the HotSpot Analysis tool (Getis-Ord Gi Statistic)^19^. The spatial unit used for georeferencing the data was the municipal name/geocode from the Brazilian Institute of Geography and Statistics (IBGE). The database of the vector layers (.shp) of the maps were obtained directly from IBGE^20^.

Temporal trends were assessed using joinpoint regression analyses to determine the annual mean percent change (AAPC) in mortality rates and to identify any significant change over time in a trend slope. The AAPC at any fixed interval was calculated using a weighted average of the specification coefficients of the underlying joinpoint regression model, with weights equal to the length of each segment over the interval. The final step of the calculation transformed the weighted average of the dependency coefficients into an annual percentage change. Joinpoint regression analysis identified the best fit for inflections in which a significant change in trend was observed using a series of permutation tests with Bonferroni correction for multiple tests. In this study, a joinpoint analysis was used to understand the effects of some variables and identify the independent variable of the years in which significant changes occurred in MRCD from 2008 to 2018 and the magnitude of these changes^21^.

This study was approved by the Research Ethics Committee of the Health Department of Bahia State on July 31, 2020. 

## RESULTS

**FIGURE 1:**
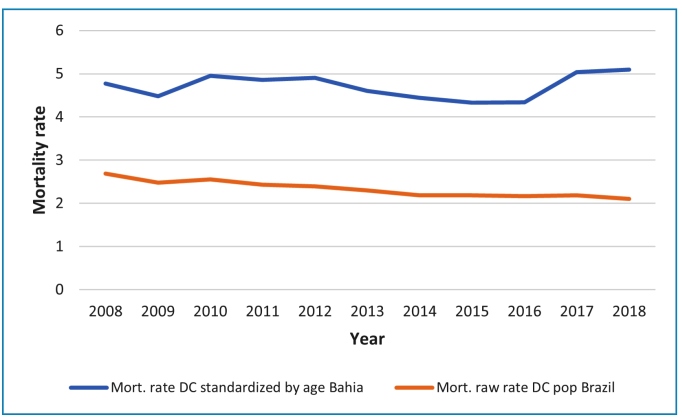
Mortality rate due to Chagas disease (primary cause), standardized by age in the state of Bahia, and gross mortality rate due to Chagas disease in Brazil, 2008-2018.

In Bahia, the Chagas disease mortality rate, age-adjusted, ranged from 4.3 to 5.1 deaths per 100,000 inhabitants between 2008 and 2018, whereas in Brazil, the rate was lower, ranging from 2.7 to 2.1 deaths per 100,000 inhabitants (Figure 1). Supplementary Figure 2 displays boxplots of MRCD by primary cause or mention of CD from 2008 to 2018 based on health macroregion. The eastern health macroregion had the highest median, followed by the Central North, Western, Central Eastern, Southwest, North, Northeast, South and Extreme South health macroregions. 

**FIGURE 2:**
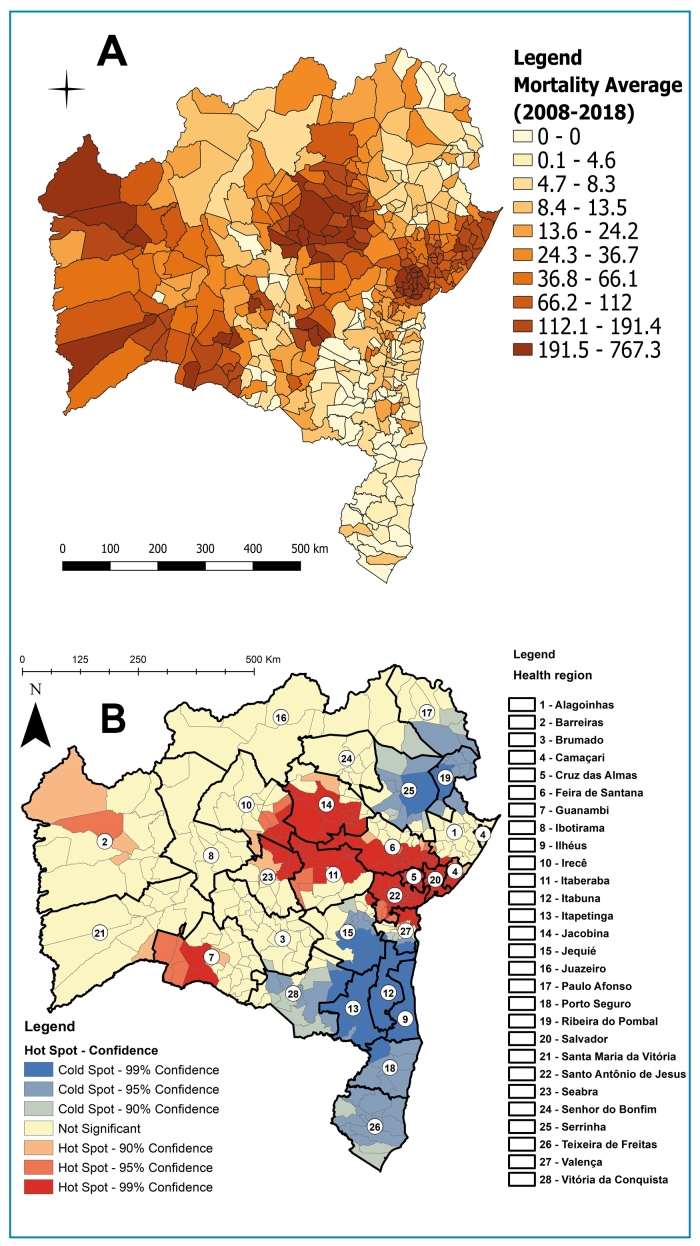
**(A).** Distribution of the mortality rate due to Chagas disease (primary cause or mention of the disease), according to municipality of residence in the state of Bahia between 2008 and 2018. (**B).** Distribution of hot and coldspots according to the mortality rate due to Chagas disease by health region and municipality of residence in Bahia, 2008-2018.

Analysis of the MRCD values revealed that areas in the east had higher coefficients (ranging from 0 to 767/100,000 inhabitants) compared to areas in the extreme south Figure 2A. The geospatial analysis of hot and coldspots (Figure 2B) revealed that hotspots were located in the health regions of the Barreiras, Jacobina, Irecê, Seabra, Itaberaba, Feira de Santana, Cruz das Almas, Santo Antônio de Jesus, and Guanambi. Coldspots were observed in several regions including Paulo Afonso, Ribeira do Pombal, Serrinha, Valença, Jequié, Vitória da Conquista, Itabuna, Ilhéus, Itapetinga, Porto Seguro, and Teixeira de Freitas. 

We compiled tables displaying deaths due to CD as the primary cause or mention of the disease, categorized according to the ICD-10 codes. Among these, 46.0% were attributed to cardiovascular diseases, 12.5% to respiratory diseases, 10.8% to neoplasms, 10.0% to digestive diseases, and 20.7% to other causes (Table 1).

**TABLE 1:** Number of deaths due to Chagas disease as the primary cause or mention of Chagas disease, according to the ICD-10 code in the state of Bahia, 2008-2018.

Chapter (CID-10)	Primary cause		CD mention	
	N	%	N	%
Infectious and parasitic diseases	7,000	99.9	85	4.7
Chapter II - Neoplasms			197	10.8
Chapter III - Endocrine, Nutritional and Metabolic Diseases			14	0.8
Chapter IV - Blood, Hematopoietic and Immune Disorders			143	7.8
Chapter V - Mental and Behavioral Disorders			12	0.7
Chapter VI - Nervous System Diseases			12	0.7
Chapter XIX - Diseases of the Circulatory System			839	46.0
Chapter X - Respiratory System Diseases			228	12.5
Chapter XI - Digestive System Diseases	4	0.1	182	10.0
Chapter XII - Diseases of the Genitourinary System			8	0.4
Chapter XIII - Osteomuscular and connective tissue diseases			8	0.4
Chapter XIV - Diseases of the Genitourinary System			55	3.0
Chapter XV - Pregnancy, Childbirth and Postpartum	5	0.1	2	0.1
Chapter XVII - Congenital Malformations, Deformities and Chromosomal Anomalies			8	0.4
Chapter XX - External Causes			32	1.8
Total	7,009	79.3	1,825	20.7

**Note:** ICD-10: International Classification of Diseases; **N**: number of deaths; **%**: percentage.

Table 2 presents the distribution of deaths based on clinical forms or complications of CD between 2008 and 2018. Among the deaths in which CD was the primary cause, 79.7% presented with the chronic form and cardiac involvement, whereas 12.0% presented with digestive system involvement. Notably, 376 deaths (5.4 %) were due to the acute form of the disease with cardiac involvement. Among the deaths with the mention of CD (1,825), 85 patients presented with more complications, totaling 1,910 mentions of the disease. Among the clinical manifestations of the disease, 1,561 had chronic cardiac involvement (83,5%) and 51 (2,7%) had acute forms of CD without cardiac involvement. Additionally, 20 deaths due to CD sequelae and seven maternal deaths with a mention of the disease were noteworthy. Four were indirect and three were late (O96: death from any obstetric cause that occurs >42 days but <1 year after birth). 

**TABLE 2:** Distribution of causes of death due to Chagas disease (primary or mentioned) according to clinical forms/complications of the disease in the state of Bahia, 2008-2018.

Clinical Form / CD complications (ICD-10)	Primary cause		CD mention	
	N	%	N	%
B57.0 - Acute form of CD with cardiac involvement	376	5.4	67	3.6
B57.1 - Acute form of CD without cardiac involvement	73	1.1	51	2.7
B57.2 - Chronic CD with cardiac involvement	5,584	79.7	1,561	83.5
B57.3 - Chronic CD with involvement of the digestive system	844	12.0	141	7.6
B57.4 - Chronic CD with nervous system impairment	34	0.5	04	0.2
B57.5 - Chronic CD with involvement of other organs	44	0.6	23	1.2
K23.1 - Mega esophagus in DC	02	0.0	04	0.2
K93-1 - Mega colon in DC	02	0.0	05	0.3
O98-6 - Diseases caused by protozoa complicating pregnancy, childbirth, and the postpartum period	03	0.0	00	0.0
O994 - Diseases of the circulatory system complicating pregnancy, childbirth, and the postpartum period	00	0.0	01	0.0
O96 - Death, from any obstetric cause, that occurs more than 42 days but less than 1 year after birth	02	0.0	01	0.0
B94.8 - Sequelae of other specified infectious and parasitic diseases	20	0.3	00	0.0
CD invalid codes - (B57, B57.X e B57.9)	25	0.4	12	0.7
**Total**	**7,009**	**100**	**1,910**	**100**

**Note: ICD-10:** International Classification of Diseases; **N**: number of deaths; **%**: percentage.

As shown in Table 3, we assessed the AAPC, 95% confidence interval (CI), and p-value considering the age group, sex, and health macroregion of residence in 2008 and 2018. The AAPC serves as a summarized measure of the trend over a specific prespecified interval, allowing the use of a single number to describe the average AAPCs during a period¹⁹. When comparing the first and last years of the data series, a trend toward variation in MRCD in Bahia was observed (AAPC: -0.2%), although this difference was not statistically significant (95% CI: -1.4; 1.1), suggesting a stationary trend. Analysis by age group revealed a progressive increase in MRCD with age. All age groups, except for the 60-69 years age group, exhibited a statistically significant increase (p<0.05), as shown in Table 3. In terms of the MRCD by sex, men had a higher mortality rate (6.7 deaths per 100, 000 inhabitants) than women. However, a reduction in coefficient was observed (AAPC: -1.2; 95% CI: -2.6; 0.2; p=0.080). In contrast, women showed an increase (AAPC: 1.3; 95% CI: 0.1; 2.5; p=0.033), with statistical significance only for women. Four of the nine health macroregions showed an upward trend in MRCD between 2008 and 2018.

**TABLE 3:** Chagas Disease Mortality Rate (MRCD), primary cause or mention of the disease, and the average AAPC in Bahia state, Brazil, 2008-2018.

Characteristics	Mortality Rate from Chagas Disease, per 100,000 inhabitants											AAPC*	95% CI**		p-value
													Min	Max	
**Age group (years)**	2008	2009	2010	2011	2012	2013	2014	2015	2016	2017	2018				
00 - 29	0.13	0.19	0.19	0.17	0.04	0.06	0.04	0.09	0.10	0.02	0.03	-10.9	-18.5	-2.6	0.017
30 - 39	1.86	1.85	2.46	1.71	1.56	1.64	1.03	1.28	1.15	0.76	0.70	-7.5	-12.1	-2.6	0.007
40 - 49	5.94	5.40	4.45	5.78	5.11	4.48	3.19	3.01	3.43	3.20	3.20	-5.4	-8	-2.7	0.002
50 - 59	17.45	13.24	15.28	12.21	13.06	13.16	9.69	9.64	8.67	11.54	11.41	-3.2	-5.6	-0.7	0.018
60 - 69	24.54	26.62	26.00	29.21	27.42	24.05	25.02	20.74	19.94	24.49	21.36	-0.1	-3.8	3.8	0.976
70 - 79	35.28	35.42	45.27	40.12	41.75	40.24	40.49	41.80	41.60	42.37	46.14	3.7	2.8	4.6	< 0,001
≥ 80	50.07	43.98	55.51	62.99	66.18	68.09	67.82	68.37	68.45	76.24	73.97	6.2	3.1	9.4	< 0,001
**Sex**															
Male	6.69	6.25	7.56	7.40	7.16	6.86	5.86	5.94	5.66	6.20	6.13	-1.2	-2.6	0.2	0.08
Female	4.03	3.98	4.71	4.68	4.71	4.52	4.48	4.11	4.19	4.73	4.57	1.3	0.1	2.5	0.033
**Health macrorregion**															
East Center	5.19	5.10	6.00	6.21	6.09	5.20	4.64	4.00	3.64	4.89	4.16	-2.5	-5.1	0.2	0.064
North Center	10.15	12.20	12.32	14.35	8.89	11.91	8.59	10.49	10.33	10.06	10.32	-1.5	-4.5	1.5	0.286
Extreme South	0.14	0.13	0.00	0.39	0.65	0.48	0.12	0.00	0.35	0.00	0.24	9.7	-8,9	32.2	0.33
East	8.84	7.70	9.83	9.11	9.54	8.55	8.44	8.37	8.35	8.23	8.01	-0.2	-1.3	0.9	0.655
Northeast	2.81	4.61	5.29	5.26	3.90	4.82	3.65	3.97	2.59	3.93	5.24	0.4	-3.8	4.9	0.833
North	1.32	0.65	1.48	2.93	2.53	3.02	2.18	2.89	2.70	3.13	1.58	-2.7	4.5	12.3	0.196
West	5.82	6.15	6.84	7.57	7.73	7.67	8.13	6.10	5.85	8.46	7.93	3.2	0.5	6	0.027
South West	3.54	3.49	3.81	2.81	3.97	3.82	2.76	2.37	3.02	4.01	5.13	3.5	-4.3	12	0.389
South	1.11	0.82	1.29	0.56	0.80	0.53	0.95	0.71	0.53	0.77	0.50	-6	-11.1	-0.7	0.032
Bahia	5.34	5.08	6.09	5.97	5.88	5.64	5.15	5.01	4.91	5.45	5.33	-0.2	-1.4	1.1	0.76

Note: *AAPC: Average Annual Percentage Change; **CI: Confidence interval.

## DISCUSSION

This study presents the temporal trends and regional differences in MRCD in Bahia State. The results indicated that CD mortality in Bahia, as the primary cause and adjusted for age, was higher than the national rate. Although a decreasing trend has been observed since 2012, the rate has increased over the last three years of the study period. These results are consistent with the findings of other studies that placed Bahia among the five states with the highest MRCD^7^ ^,^ ^22^. Between 2006 and 2011, the Federation Units with the highest MRCD were observed in Goiás (12.2/100,000 inhabitants), the Federal District (7.9/100,000 inhabitants), Minas Gerais (6.4/100,000 inhabitants), Bahia (4.5/100,000 inhabitants), and Tocantins (3.7/100,000 inhabitants)^22^. 

The CD scenario is dynamic, and individuals with this disease require comprehensive and articulated care^23^. Therefore, we suggest implementing public policies starting at the health-region level and identifying priority municipalities and areas for state-level interventions.

In Bahia, we identified hotspots for CD mortality, particularly in the Barreiras, Guanambi, Irecê, Itaberaba, Santa Maria da Vitória, and Santo Antônio de Jesus health regions. These findings confirm the considerable impact of CD in Bahia and highlight priority areas for planning and implementing prevention, health surveillance, and vector control measures, with a focus on areas with the highest MRCD. We also observed that four of the nine health macroregions showed an upward trend in MRCD between 2008 and 2018. Of these, CD is historically endemic to only two regions (West and Southwest)^9^.

Organized vector control has been implemented since 1975, achieving total coverage of the endemic area in 1983, which explains the generation of individuals with CD who live in areas of intense vector transmission. However, the Brazilian Ministry of Health has warned that these control actions, previously carried out by the Federal Government, have progressively decreased since the late 1980s, which could compromise the success of CD control^24^. 

In Bahia State, we observed that several municipalities presented high vulnerability^11^, explained by the high occurrence of synanthropic species of triatomines living near poor people in rural areas^25^. However, only two acute cases of CD were reported during the study period. Notably, in Bahia, a high percentage of houses are located in rural areas with people living in extreme poverty, and the absence of reported cases may be because of the low sensitivity of detecting acute CD cases^26^.

Regarding the characteristics of individuals who died from CD during the study period, we observed a progressive increase in mortality with age, consistent with the findings of other studies conducted in Brazil and Colombia, where older age groups were associated with MRCD^7^ ^,^ ^23^ ^,^ ^27^ ^,^ ^28^. However, the current increase in life expectancy among Brazilians is not accompanied by improved health conditions. This hypothesis was confirmed by Bonfim^29^, who addressed healthy life expectancy in older individuals in Bahia and the Northeast using data from the Brazilian National Health Survey and demonstrated a reduction in healthy life expectancy and worsening living conditions over the years. This finding suggests that older individuals with CD are likely to live with comorbidities that directly impact their quality of life and the health care system. 

Studies on older individuals with CD investigating the association between clinical forms of the disease and chronic conditions found that older individuals with CD are particularly vulnerable to the harmful effects of the combination of CD and chronic degenerative diseases, which are exacerbated by the polypharmacy they use^30^.

Additionally, CD-related mortality was higher among men than among women and increased with age, both as the primary cause of death and as mentioned in the death certificate. Although a tendency to reduce MRCD in men was observed, in the period from 2008 to 2018, this rate was higher than that among women, corroborating other studies^7^ ^,^ ^22^ ^,^ ^27^ ^,^ ^28^ that also showed a predominance of mortality due to CD among men. Therefore, health surveillance is needed to increase the suspicion of CD in men. Although women had lower MRCD in the present study, we observed an increase in this rate in the years studied, recommending a careful assessment of the future trend of this coefficient among women, as well as the inclusion of actions to prevent vertical transmission of CD in the prenatal period.

Although Bahia State reported only two laboratory-confirmed cases of acute CD in the Notifiable Diseases Information System during the study period, we observed a significant number of deaths from acute CD, either as a primary cause or as mentioned on death certificates. Given that these records were not investigated, we cannot guarantee the accuracy of this information as acute cases; however, as these have been registered in the SIM with the international disease code, the diagnosis of CD is highly probable. 

We found that a substantial number of death certificates mentioned CD as a cause of circulatory system disease (46%). Considering that the main complication of the disease is cardiac involvement, some of these deaths may have been due to CD complications. Therefore, this classification may have been incorrect. Regarding clinical forms and complications, most deaths were associated with CD, with cardiac involvement as the primary cause, followed by digestive system involvement, which is consistent with the findings of other studies^7^ ^,^ ^22^ ^,^ ^29^ ^,^ ^30^. Notably, deaths classified as resulting from sequelae may be mistaken, as there is a possibility that they are chronic cases of the disease. According to ICD 10, the classification of sequelae is used to indicate conditions that are not present at the moment, but are the cause of the current problem, which is under treatment or investigation. Thus, to be considered sequelae, the causal condition must not be present at the moment^15^. As there is no recommendation to perform serology to monitor the cure of chronic patients, it is possible that the information that the cases reported in the SIM are sequelae would need to be investigated.

Deaths were also reported with invalid codes, which have a high chance of being added to deaths due to chronic CD with cardiac involvement, the most frequent form observed in studies^7^ ^,^ ^22^ ^,^ ^29^ ^,^ ^30^. We identified a representative number of deaths in the acute phase of the disease (approximately 7% of the study records). We believe that there is a possible underreporting of the disease in the acute phase, since it is expected that approximately 1% of deaths due to CD in the acute phase evolve to deaths, which indicates possible inaccuracies in the clinical classification of the disease in death certificates, reaffirming the need for training of the health team.

Another major finding of this study was the seven maternal deaths associated with cardiac involvement, emphasizing the preventability of these deaths through prevention and control measures. Screening for CD in pregnant women from endemic areas of Bahia is recommended^31^. Timely diagnosis provides adequate care for both the mother and baby. Healthcare teams must consider these guidelines, particularly in vulnerable regions^11^. Healthcare leaders must provide a structure for healthcare teams to offer disease prevention, entomological surveillance, and comprehensive care to patients with CD, in addition to medium- and high-complexity services for cases of the disease that need this support.

Because the death certificate is essential for understanding the cause and monitoring the trend of the disease in a given area and period, the need to invest in continuing education for the healthcare and surveillance network, qualifying the care of patients with CD, and the registration, classification, and processing of information is necessary. Recent research^32^ suggests that institute training for medical teams on filling out death certificates is essential to make these professionals aware of the relevance of this document for public policies.

This study identified and discussed the magnitude of CD in Bahia by analyzing the MRCD and delineating priority areas and populations based on this indicator. These results can inform the planning of prevention, control, and follow-up actions for patients with CD in priority areas and vulnerable populations. 

Our results also showed that CD mortality in Bahia State do not follow a homogeneous pattern, indicating that the most vulnerable regions are those in the east, with a higher MRCD compared to regions in the south, coinciding with risky areas of *T. cruzi* vector transmission^8^ ^,^ ^11^. Therefore, the regional differences observed require local planning reorganization, prioritization, and integration of epidemiological and entomological surveillance in healthcare. This challenge is present in all areas with NTD and must be addressed at each level of management to protect vulnerable populations from the risk of acquiring the disease.

This study had several limitations. Notably, secondary data from the Brazilian SIM was used, which, although covering 100% of all municipalities, has deficiencies in the quality of some information. We excluded 28 death records from the georeferencing analysis that did not include information on the municipality of residence. Additionally, 25 invalid codes for the underlying cause or mention of CD were identified, making it impossible to determine the clinical form of these cases.

Another limitation of this study was the analysis of the causes of death. The clinical form of death due to CD with the highest incidence was chronic with cardiac involvement (ICD-10: B57.2). However, this ICD includes not only chronic CD with cardiac involvement but also chronic CD not otherwise specified (NOS) and CD NOS in settings where it is prevalent, potentially compromising this analysis. In ICD-10, no code exists for the undetermined chronic form of CD; only codes for unspecified causes of chronic CD and chronic CD with cardiac involvement are included, which have the same code, B57.2^15^. Although most cases recorded by this code may refer to chronic CD with cardiac involvement, attention should be paid to the descriptions covered by this code. However, the ICD-11 includes a specific code for the indeterminate chronic form of CD^33^.

Despite these limitations, regional and individual differences in MRCD, as noted in this study, can support health planning to address regional needs. Four of the nine health macroregions displayed rising MRCD, underscoring the necessity for expanded access to quality care for affected individuals. Continuous monitoring and proactive healthcare measures are crucial for preventing severe outcomes and premature mortality. Sustained efforts are vital for preventing further CD transmission.
