# Supplementary material for: Spatio-temporal trends in mortality due to Chagas disease in the State of Bahia, Brazil, from 2008 to 2018
Source: Rev Soc Bras Med Trop. 2024 Oct 28;57:e00417-2024. doi: 10.1590/0037-8682-0058-2024 (PMC11524596; doi:10.1590/0037-8682-0058-2024)
Supplement: Supplementary file 1 [file 1678-9849-rsbmt-57-e00417-2024-supp1.pdf]

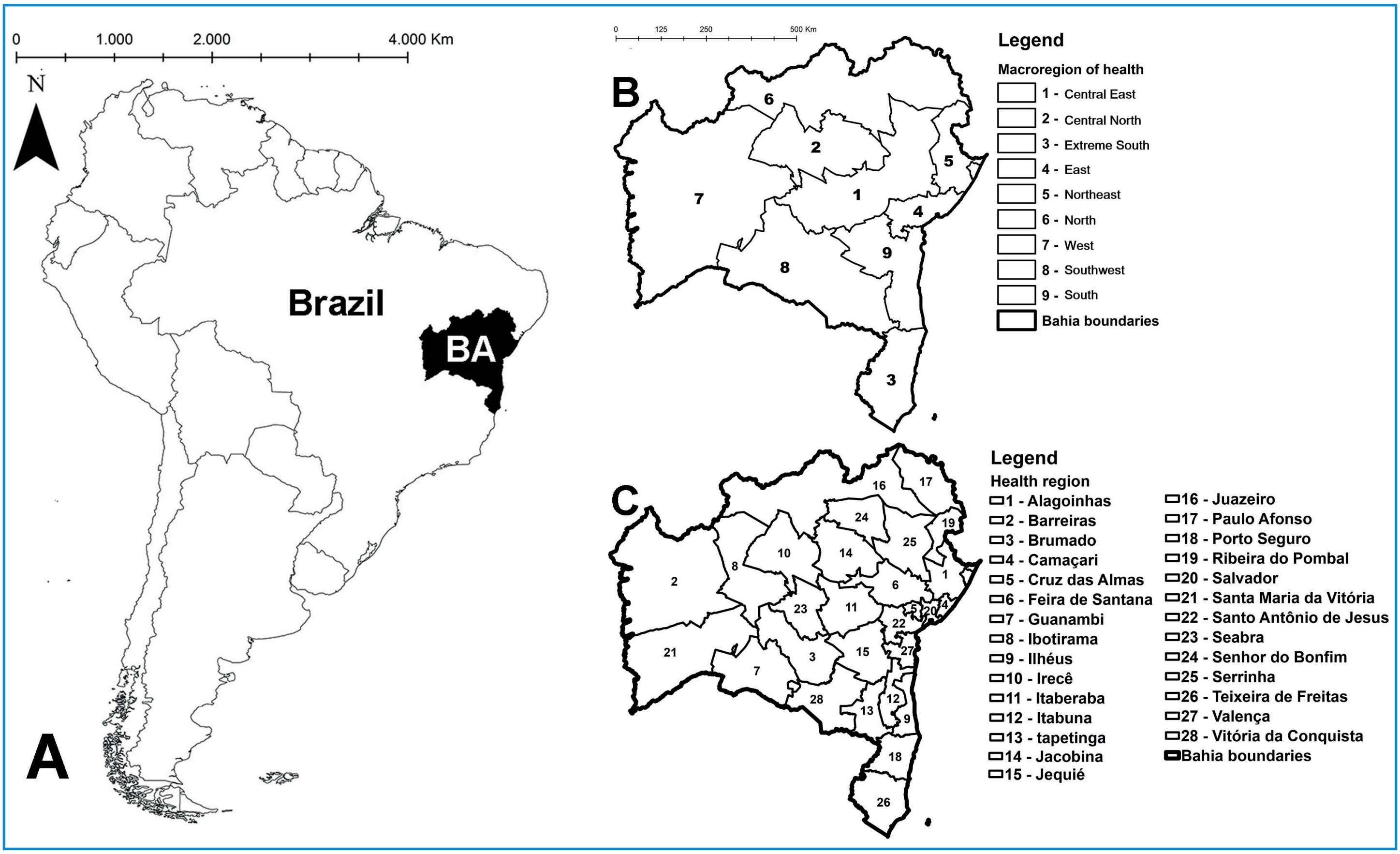

SUPPLEMENTARY FIGURE 1: Study area. **(A).** South American countries, Brazil, and in detail, the state of Bahia. Geopolitical delimitations of health macroregions **(B)** and health regions **(C).**
